# Microstructure evolution, dielectric properties, and nonlinear response of Na^+^-doped CdCu_3_Ti_4_O_12_ ceramics

**DOI:** 10.1039/d4ra04602a

**Published:** 2024-08-16

**Authors:** Renzhong Xue, Xiaosong Liu, Kun Yang, Xiang Zhu

**Affiliations:** a School of Electronics and Information, Zhengzhou University of Light Industry Zhengzhou 450002 PR China xrzbotao@163.com; b Henan Key Laboratory of Magnetoelectronic Information Functional Materials, Zhengzhou University of Light Industry Zhengzhou 450002 PR China

## Abstract

In this study, Cd_1−*x*_Na_*x*_Cu_3_Ti_4_O_12_ (*x* = 0, 0.02, 0.04, 0.06, and 0.08) ceramics were prepared *via* a solid-state method. The phase composition, microstructure, and defect characteristics as well as optical, dielectric, and nonlinear properties of the ceramics were systematically studied. A CuO second phase was detected in doped samples. Grain boundary precipitates, Na with a low melting point, and oxygen and cation vacancies together caused the grain size to first increase and then decrease with an increase in the Na^+^ doping amount. The abundant emerging cation vacancies with an increase in Na^+^ content led to a decrease in the optical energy band. The sample with *x* = 0.04 exhibited the highest *ε*′ value (∼35 800) due to its largest grain size. Moreover, it possessed a lower tan *δ* (∼0.053) at 10 kHz, which was attributed to the multiplication of insulating grain boundaries. The huge dielectric constant originated from Maxwell–Wagner polarization at low frequencies and followed the internal barrier effect model. The lowest tan *δ* (∼0.037) and optimal nonlinear properties (*α* = 3.66 and *E*_b_ = 3.82 kV cm^−1^) were obtained in the sample with *x* = 0.08, which were associated with its highest grain boundary resistance and barrier height. Electric modulus data proved that dielectric relaxation at low frequencies was associated with grain boundaries. Dielectric anomalies in the high temperature range were attributed to oxygen vacancies.

## Abstract 2

The nonlinear current density–electric field (*J*–*E*) plots of Cd_1−*x*_Na_*x*_Cu_3_Ti_4_O_12_ ceramics. All samples exhibited good nonlinear characteristics.

## Graphical abstract

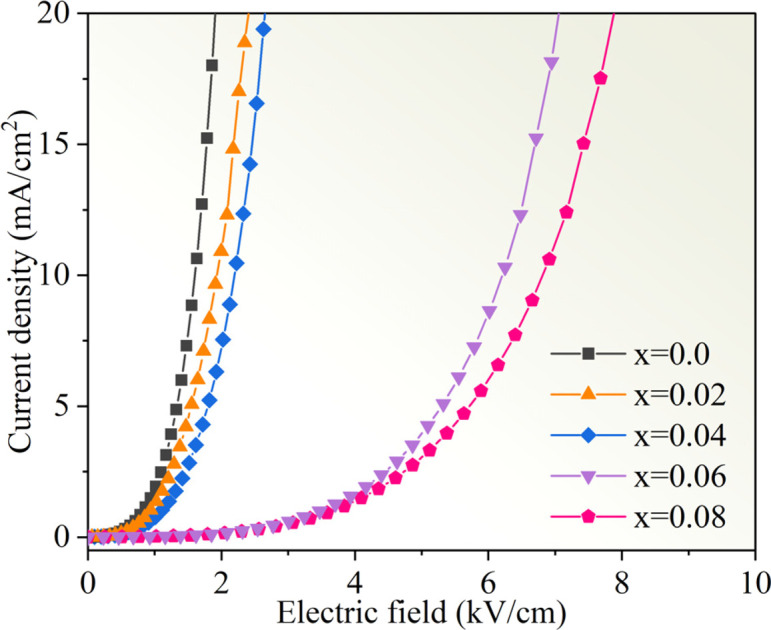

## 1. Introduction

CaCu_3_Ti_4_O_12_ (CCTO) has a high dielectric constant (10^4^) at low frequencies, which remains stable in the temperature range from 100 to 600 K.^1–3^ This peculiarity makes it able to increase the storage capacity per unit volume and reduce the size of the device, which is of great importance in dynamic memory, 5G communication, and energy storage fields.^4,5^ Unlike other perovskite materials, the high dielectric constant of CCTO is not due to the dipole polarization mechanism but due to the Maxwell–Wagner polarization based on semiconducting grains and insulating grain boundaries.^3,6,7^ With the continuous progress in research, such materials have formed a large ACu_3_Ti_4_O_12_ family (ACTO, A = Ca, Cd, Bi_2/3_, Y_2/3_, La_2/3_, *etc.*).^1,8–11^ However, in this type of material, a higher dielectric constant (*ε*′) usually means a greater dielectric loss (tan *δ*). Therefore, exploring the origin of the large dielectric constant and searching for possibilities to increase dielectric constant while maintaining low dielectric loss is an urgent task. Notably, owing to their electrically heterogeneous structure and high barrier, grain boundaries produce nonlinear current–voltage properties, which are appealing for varistors.^12–14^

CdCu_3_Ti_4_O_12_ (CdCTO) is a member of ACTO-type materials. The first achieved dielectric constant of CdCTO was only 409 at 10^5^ Hz.^1^ After improving the preparation process, a large dielectric constant (10^4^) could be attained.^15,16^ Subsequently, Mg^2+^, Zn^2+^, Zr^4+^, and other elements were used as dopants in CdCTO.^17–21^ Peng *et al.* reported high *ε*′ values (>4 × 10^4^) at a relatively low tan *δ* (<0.1) at 1 kHz in CdCu_2.9_Zn_0.1_Ti_4_O_12_ and CdMg_0.1_Cu_2.9_Ti_4_O_12_ ceramics.^17,18^ Nonlinear coefficient (*α*) and breakdown field strength (*E*_b_) are two very important parameters of varistors. Peng *et al.* observed an improvement in both parameters from *α* ∼3.15 and *E*_b_ ∼0.257 kV cm^−1^ for CdCTO to *α* ∼4.98 and *E*_b_ ∼1.78 kV cm^−1^ for 3.0 wt% Al_2_O_3_-doped CdCTO ceramics and to *E*_b_ ∼2.36 kV cm^−1^ for 4.0 wt% SiO_2_-doped CdCTO ceramics, respectively, although the additives led to a decrease in dielectric constant.^20,21^ In general, the current research on the dielectric properties of CdCTO is still at its initial stage, especially in terms of the nonlinear properties. Therefore, the development of advanced methods to optimize the dielectric and nonlinear properties is essential for large-scale applications of CdCTO materials.

Ion doping or substitution is one of the important means to improve the ACTO properties. Among them, Na^+^ doping of A-sites in ACTO has been reported. In Na^+^-doped La_2/3_Cu_3_Ti_4_O_12_ and Y_2/3_Cu_3_Ti_4_O_12_ ceramics, the grain size varied in an unpredictable manner, while increasing continuously in Bi_2/3_Cu_3_Ti_4_O_12_ and CaCu_3_Ti_4_O_12_ ceramics.^22–25^ The optimal dielectric properties (*ε*′ ∼97 647 and tan *δ* ∼0.073) were attained in the Ca_0.98_Na_0.02_Cu_3_Ti_4_O_12_ ceramic, exceeding those of the Na_0.35_Bi_0.55_Cu_3_Ti_4_O_12_ (*ε*′ ∼7600 and tan *δ* ∼0.015), Na_0.05_Y_0.65_Cu_3_Ti_4_O_12_ (*ε*′ ∼7500 and tan *δ* ∼0.022), and Na_0.5_La_0.5_Cu_3_Ti_4_O_12_ (*ε*′ ∼15 000 and tan *δ* ∼0.047) ceramics, respectively.^22–25^ Meanwhile, the effects of Na^+^ on the microstructure and dielectric properties of the above materials were poorly understood, in spite of the fact that doping generally improves the dielectric properties. This meant that the underlying mechanism of doping in relation to performance was unclear. Moreover, the nonlinear electrical properties of these materials were not explored. In addition, very few studies have been focused on CdCTO ceramics with different proportions of Na^+^ doping. In this work, Cd_1−*x*_Na_*x*_Cu_3_Ti_4_O_12_ (*x* = 0, 0.02, 0.04, 0.06, and 0.08) ceramics were prepared *via* solid-state reaction method. The effect of Na^+^ doping on the microstructure, optical and dielectric characteristics, complex impedance behavior, and nonlinear properties of ceramics was systematically investigated. The optimized dielectric and nonlinear properties were shown to provide more options for the application of ACTO materials. The relevant mechanism affecting the microstructure of Cd_1−*x*_Na_*x*_Cu_3_Ti_4_O_12_ ceramics may be fundamental for further clarification of the origin of the large dielectric response in ACTO ceramics.

## 2. Materials and methods

### 2.1. Powders and ceramics preparation

Cd_1−*x*_Na_*x*_Cu_3_Ti_4_O_12_ (*x* = 0, 0.02, 0.04, 0.06, and 0.08) ceramics were prepared *via* solid-state method. The starting materials were CdO (99%), NaCO_3_ (99.99%), CuO (99%), and TiO_2_ (99%) (all purchased from Shanghai Aladdin Biochemical Technology Co., Ltd). Precursors were mixed proportionally, and ground with an agate mortar for 3 h. The obtained powders were afterward calcined at 800 °C for 10 h, ground for 3 h with the addition of 2 wt% PVA, and pressed into disk-shaped pellets with 10 mm diameter and 1 mm thickness at 8 MPa. Pellets were first sintered at 550 °C for 2 h to remove PVA, and then at 1000 °C for 15 h in air at the heating rate of 3 °C min^−1^. Prior to electric measurements, both sides of specimens were coated with silver paste and heated at 300 °C for 20 min.

### 2.2. Structural and electrical property characterization

The crystal structure and phase composition of specimens were analyzed *via* X-ray diffraction (XRD, SmartLab, Rigaku, Japan; Cu-Kα radiation (*λ* = 1.5418 Å)) and Raman spectroscopy (Renishaw InVia, UK; 532 nm laser excitation wavelength). The Rietveld refinement of XRD profiles was performed in the GSAS software. The densities of specimens were assessed *via* Archimedes' method. The microstructure and elemental compositions of ceramics were characterized *via* field emission scanning electron microscopy (FE-SEM, JSM-7000F, Japan). The average grain size was determined from the FE-SEM images using the Nano Measurer software. The overall chemical composition was determined from inductively coupled plasma mass spectroscopy (ICPMS, Agilent 7700, USA). Dried Cd_1−*x*_Na_*x*_Cu_3_Ti_4_O_12_ starting powders and sintered ceramic powders of 30–50 mg were dissolved in 25 ml of an *aqua regia* and hydrofluoric acid mixture, and diluted with deionized water by 1 and 100 times, respectively, for Na and other elements prior to analysis. The positron annihilation lifetime spectra were recorded to establish the defect characteristics by means of a fast–fast coincidence lifetime spectrometer (ORTEC, USA); prior to the experiment, a ^22^Na positron source was sandwiched between two specimens of the same composition. During the measurements, more than 10^6^ counts were collected. The PALS fit software was used for data processing. Ultraviolet-visible (UV-vis) spectroscopy (UH4150, China) was employed to obtain the absorption characteristics of the specimens. The dielectric and complex impedance spectra of ceramics were measured with a precision impedance analyzer (Agilent 4294A, USA). The current density–electric field (*J*–*E*) characteristics were acquired with the Keithley 2400 test system. The nonlinearity coefficients *α* were calculated according to the following formula: *α* = log(*J*_2_/*J*_1_)/log(*E*_2_/*E*_1_), where *E*_1_ and *E*_2_ are the voltages at currents *J*_1_ = 0.1 mA and *J*_2_ = 1 mA, respectively. The *E*_b_ values were obtained at *J*_1_ = 1 mA cm^−2^.

### 2.3. First-principles calculation details

The first-principles calculations were carried out to evaluate the stability of Cd_1−*x*_Na_*x*_Cu_3_Ti_4_O_12_ crystal structures by using the Cambridge Serial Total Energy Package code.^26^ The interactions between electrons and ionic nuclei were modeled by means of Vanderbilt-type ultrasoft pseudopotentials so as to establish the electronic structure of the specimen.^27^ The generalized gradient approximation based on the Perdew–Burke–Ernzerhof functional was performed to evaluate the exchange correlation energy.^28^ The energy of the first Brillouin zone was calculated using a 4 × 4 × 4 *K*-point grid in Monkhorst–Pack format, whereby the cutoff energy of the plane wave base set of the electron wave function was 340 eV.

## 3. Results and discussion

**Fig. 1:**
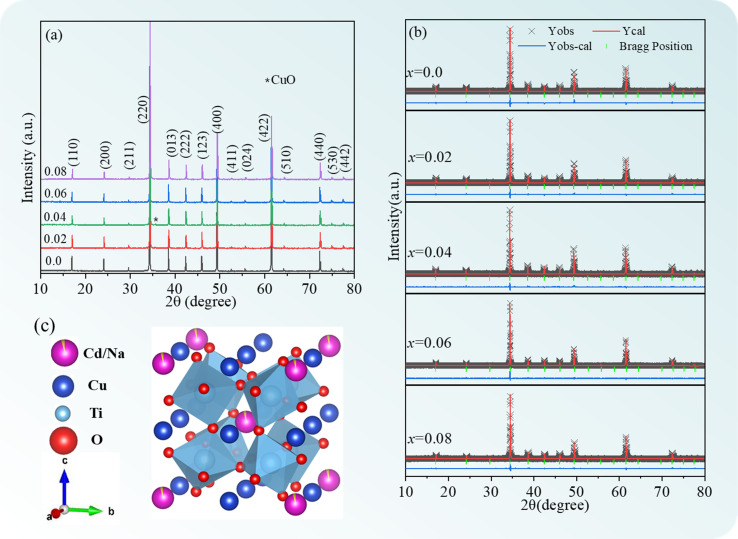
(a) XRD patterns of Cd_1−*x*_Na_*x*_Cu_3_Ti_4_O_12_ ceramics; Rietveld refinement plots in the case of (b) CdCTO and (c) BCTO; (d) crystal structure of Cd_1−*x*_Na_*x*_Cu_3_Ti_4_O_12_.

Fig. 1(a) depicts the XRD patterns of the Cd_1−*x*_Na_*x*_Cu_3_Ti_4_O_12_ ceramics. The major diffraction peaks were ascribed to the body-centered cubic perovskite (JCPDS card no. 75-2188), corresponding to the CCTO phase. A CuO second phase appeared in the samples with *x* = 0.02 and 0.04. The lattice distortion was caused by the substitution of Na^+^ ions (*r*_6_ = 1.02 Å) for Cd^2+^ (*r*_6_ = 0.95 Å),^29^ which promoted the formation of a Cu-rich grain boundary layer.^23,30^ According to the CaO–CuO–TiO_2_ ternary system, the compound CCTO appeared to be a “point compound” with very narrow solubility limits.^31^ When the composition deviated slightly from perfect stoichiometry, the material ended up along a binary tie-line or inside a ternary field, resulting in the second phases in the microstructure of CCTO.^31^ This resulted in the precipitation of the CuO phase in the ACTO material.^31,32^ Similar results were also reported in the CdO–CuO–TiO_2_ ternary system.^33^ The CuO second phase observed in the samples with *x* = 0.02 and 0.04 should be closely related to the Cd site component deviation caused by Na doping. The Rietveld refinement of the XRD spectra was further carried out to obtain detailed information about the lattice parameters of the Cd_1−*x*_Na_2*x*_Cu_3_Ti_4_O_12_ ceramics (the results are displayed in Table 1 and Fig. 1(b)). It was evident that the simulated curves effectively matched the experimental data, indicating decent reliability of the refinement. The unit cell parameter of CdCTO was found to be 7.3839 Å, and coincided with the value reported in ref. 15. This parameter increased in specimens with *x* = 0.02 and 0.04, owing to the larger ionic radius of the dopant.^29^ This meant that Na^+^ was successfully incorporated in the Cd sites of the CdCTO ceramics, forming a crystal structure of Cd_1−*x*_Na_2*x*_Cu_3_Ti_4_O_12_, which is presented in Fig. 1(d). However, the unit cell parameter value decreased at *x* = 0.06 and 0.08. This is likely due to the limit of the Na solubility in Cd sites, and will be discussed later.

**Table 1:** Structural data and relative densities of Cd_1−*x*_Na_*x*_Cu_3_Ti_4_O_12_ ceramics

Samples	0.0	0.02	0.04	0.06	0.08
Space group	*Im*3̄	*Im*3̄	*Im*3̄	*Im*3̄	*Im*3̄
Unit cell parameters (Å)	*a* = *b* = *c* 7.3823(6)	*a* = *b* = *c* 7.3918(2)	*a* = *b* = *c* 7.3921(2)	*a* = *b* = *c* 7.3915(5)	*a* = *b* = *c* 7.3815(3)
Volume (Å^3^)	402.323	403.878	403.928	403.829	402.192
*R*-Factors (%)	*R*_p_ = 6.19	*R*_p_ = 5.69	*R*_p_ = 5.33	*R*_p_ = 4.89	*R*_p_ = 5.44
	*R*_wp_ = 8.04	*R*_wp_ = 7.34	*R*_wp_ = 6.49	*R*_wp_ = 6.16	*R*_wp_ = 6.97
Relative density (%)	*χ* ^2^ = 2.015	*χ* ^2^ = 1.649	*χ* ^2^ = 1.465	*χ* ^2^ = 1.235	*χ* ^2^ = 1.575
	93.9	94.5	95.8	95.2	95.1

**Fig. 2:**
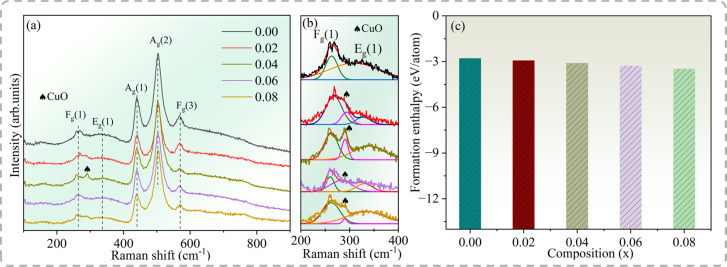
(a) Raman spectra, (b) the deconvoluted Raman spectra and (c) formation enthalpy of the Cd_1−*x*_Na_*x*_Cu_3_Ti_4_O_12_ ceramics determined *via* first-principles calculations.

The Raman spectra were collected to reveal the effect of Na^+^ doping on the Cd_1−*x*_Na_2*x*_Cu_3_Ti_4_O_12_ ceramics. Because of the weak scattering of ACTO, only a few of the eight modes (2A_g_ + 2E_g_ + 4F_g_) allowed by the selection rules appeared in the spectra.^34^Fig. 2(a) displays the Raman spectra of the Cd_1−*x*_Na_*x*_Cu_3_Ti_4_O_12_ ceramics in the wavenumber range of 100–900 cm^−1^, revealing the bands at 265, 330, 438, 506 and 571 cm^−1^. Among them, the peaks at 265, 330, 438 and 506 cm^−1^ correspond to F_g_(1), E_g_(1), A_g_(1) and A_g_(2) vibration modes originating from the TiO_6_ rotation, while the feature at 571 cm^−1^ was attributed to the F_g_(3) vibration mode related to the O–Ti–O anti-stretching atomic motion in the TiO_6_ octahedron.^34^ The results are similar to those reported in ref. 8–20. The positions of three main peaks (E_g_(1), A_g_(1), and A_g_(2)) remained unchanged with Na^+^ doping, which meant that Na^+^ had no effect on either the charge distribution or the Ti–O vibration in the TiO_6_ octahedron. Remarkably, the mode at 292 cm^−1^ at *x* = 0.04 was ascribed to the CuO phase,^35^ which was consistent with the XRD results. To further clarify the mode of the CuO phase in the specimens, Raman spectra were deconvoluted in the wavenumber range of 200–400 cm^−1^, as shown in Fig. 2(b). The results confirmed the existence of the CuO phase in other doped samples.

The formation enthalpy can reflect the thermodynamic stability of the crystal structure. Based on the first-principles calculation, the formation enthalpy of the Cd_1−*x*_Na_*x*_Cu_3_Ti_4_O_12_ ceramics can be obtained as follows:^36^1
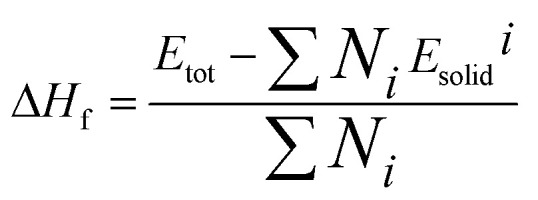
where Δ*H*_f_, *E*_tot_, *N*_*i*_, and *E*_solid_^*i*^ are the formation enthalpy, the total energy of the unit cell, the number of *i*-th atoms in the unit cell, and the total energy of each atom of the pure element in its ground state, respectively. Fig. 2(c) depicts the formation enthalpy of the Cd_1−*x*_Na_*x*_Cu_3_Ti_4_O_12_ ceramics. The negative value of CdCTO indicates its stable structure. With the increase of Na^+^ doping, the Δ*H*_f_ value tended to be more negative. As is known, the stability of a crystal structure increases with the increase of the negativity of the formation enthalpy.^36^ Thus, this increased negative Δ*H*_f_ value indicated the increase in structural stability of the doped samples.

**Fig. 3:**
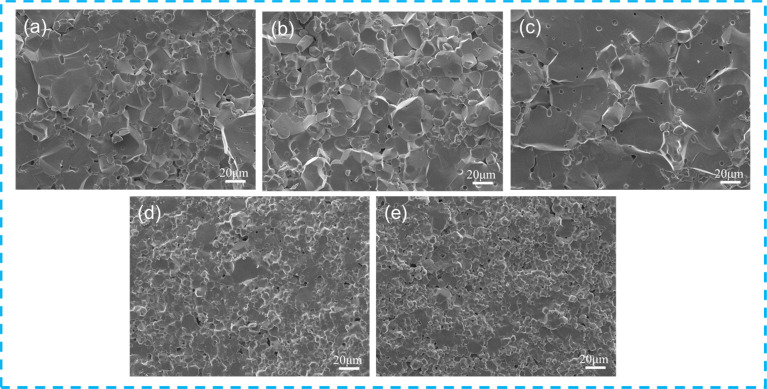
Cross-sectional SEM images of Cd_1−*x*_Na_*x*_Cu_3_Ti_4_O_12_ ceramics: (a) *x* = 0; (b) *x* = 0.02; (c) *x* = 0.04; (d) *x* = 0.06; (e) *x* = 0.08.

Fig. 3 shows the cross-sectional SEM images of the Cd_1−*x*_Na_*x*_Cu_3_Ti_4_O_12_ ceramics. The abnormally large grains and scattered small grains with scarce pores were clearly observed in CdCTO (Fig. 3(a)). The grain size increased with the increase of the Na^+^ doping content, achieving a maximum in the sample with *x* = 0.04. Notably, the increase in Na^+^ doping content caused a dramatic decrease in grain size. The average grain sizes at *x* = 0, 0.02, 0.04, 0.06, and 0.08 were 16.06 ± 3.12, 16.35 ± 3.03, 21.78 ± 4.22, 8.19 ± 1.14, and 6.37 ± 0.58 μm, respectively. The relative densities of the specimens are listed in Table 1, all exceeding 93% and indicating a dense structure.

**Fig. 4:**
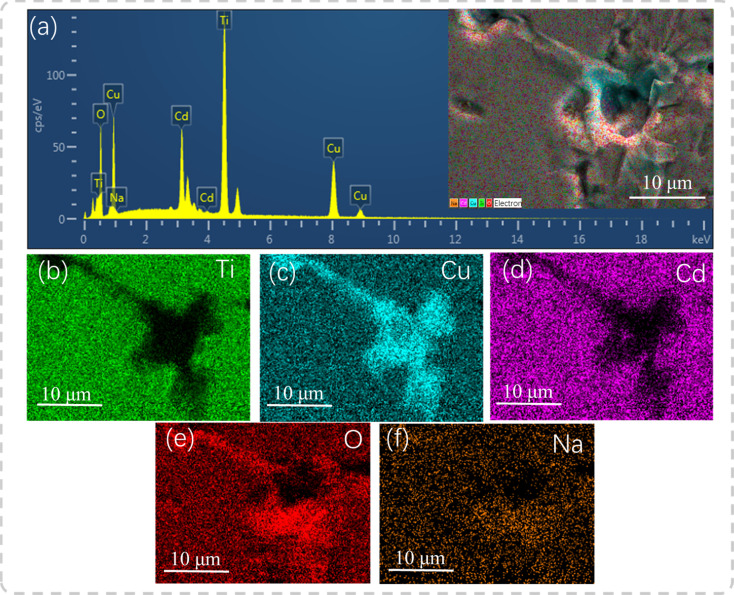
(a) EDS spectrum at *x* = 0.04; the inset shows the polished and thermally etched surface of the sample; (b–f) elemental maps.

EDS was used to reveal the element distribution in the samples. Fig. 4(a) depicts the EDS spectrum of the polished and thermally etched surface of the sample with *x* = 0.04 (see the inset), in which all elements were observed. Fig. 4(b–f) display the corresponding elemental maps, showing that Ti and Cd elements were prevalent in the grain region, but were scarce at the GBs. In turn, the Na elements were uniformly distributed, and the CuO phase was abundant at the GBs. This is similar to the data acquired on the Na^+^-doped CCTO ceramics in study.^22^ In the ACTO-like materials, the abnormal grain growth is usually associated with the presence of the CuO phase at the grain boundaries as a sintering aid during the high temperature treatment.^7,9,37–39^ Similarly, Na_2_CO_3_ with a low melting point (∼850 °C) also acted as the source of the liquid phase and promoted the grain growth.^22,23^ In addition, due to the unbalanced charge between Cd^2+^ and Na^+^, oxygen vacancies were created, conforming to the reaction below:2
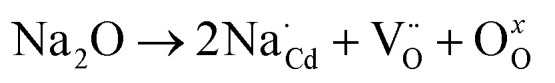


The oxygen vacancies promoted the grain boundary migration during the sintering process and increased the grain size. However, the grain size clearly decreased in the specimens with *x* = 0.06 and 0.08. This grain refinement might be due to excessive Na^+^ doping by analogy with that observed in the Na_*x*_La_(2−*x*)/3_Cu_3_Ti_4_O_12_ and Na_*x*_Y_(2−*x*)/3_Cu_3_Ti_4_O_12_ ceramics,^23,25^ which was not further interpreted. It was assumed that the intensive volatilization of Na with the low melting point in the sintering process might have produced the cation vacancies and inhibited the grain growth. To clarify the percentage for the chemical composition in the precursors and the sintered powders, ICPMS measurements were performed. The results are displayed in Table 2. The proportions of individual elements in the precursors (shown in parentheses) were consistent with those of Cd_1−*x*_Na_*x*_Cu_3_Ti_4_O_12_. However, the proportions of Na were lower in the sintered powders, corresponding to the volatilization during the sintering process. Nevertheless, the Na content in the sintered samples increased with the increase of doping amount. Combined with the above reduced cell parameters in the samples with *x* = 0.06 and 0.08, it indicated that the solubility limit of Na in the Cd sites was exceeded. According to the phase diagram of the CdO–CuO–TiO_2_ system, the Cu-rich and Ti-rich phase existed at the grain boundary in Cd_*x*_Cu_3_Ti_4_O_12_ (*x* < 1) ceramics.^33^ Thus, a binary Na_2_O–TiO_2_ compound might incorporate all of the Na preferentially in the specimens with *x* = 0.06 and 0.08, which are located at the grain boundary and had a higher melting point to inhibit the grain growth.^40^ These second phases were not detected in the above XRD patterns and Raman spectra due to their small amount.

**Table 2:** Percentage for the chemical composition of the sintered powders (the precursors) determined *via* ICPMS

Samples	Element content (at mol%)			
	Na	Cd	Cu	Ti
*x* = 0.0	0(0)	4.96(5.01)	14.94(15.02)	21.12(20.04)
*x* = 0.02	0.06(0.09)	4.83(4.88)	14.96(15.05)	21.10(20.06)
*x* = 0.04	0.12(0.18)	4.71(4.75)	14.97(15.02)	21.14(20.11)
*x* = 0.06	0.18(0.28)	4.61(4.64)	14.95(15.02)	21.16(20.09)
*x* = 0.08	0.23(0.37)	4.48(4.52)	14.96(15.04)	21.18(20.05)

**Fig. 5:**
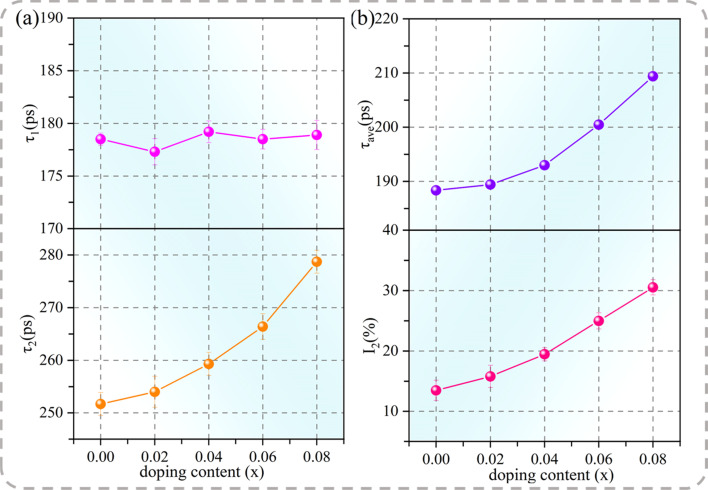
(a) Positron lifetime components, *τ*_1_ and *τ*_2_; (b) *τ*_ave_ and *I*_2_ as functions of the Na^+^ doping content in Cd_1−*x*_Na_*x*_Cu_3_Ti_4_O_12_ ceramics.

The positron annihilation technique was employed to describe the cation vacancy characteristics of the specimens. In previous studies, the annihilation process within the ACTO ceramics has been interpreted in the context of the standard two-state trapping model.^6,9,41^ In this model, a short lifetime component *τ*_1_ is related to the positron annihilation in the bulk. Meanwhile, a long lifetime component *τ*_2_ represents the positron annihilation at the cation vacancy, where the electron density declines because of missing ions.^42^ The intensity *I*_2_ indicates the concentration of defects. In addition, the average lifetime (*τ*_ave_ = *τ*_1_*I*_1_ + *τ*_2_*I*_2_) is a more reliable parameter reflecting the defect content. The *τ*_1_, *τ*_2_, *I*_1_, *I*_2_, and *τ*_ave_ values of the Cd_1−*x*_Na_*x*_Cu_3_Ti_4_O_12_ ceramics, obtained from the lifetime spectra, are listed in Table 3. Fig. 5 depicts the dependences of *τ*_1_, *τ*_2_, *τ*_ave_, and *I*_2_ on the Na^+^ content in the Cd_1−*x*_Na_*x*_Cu_3_Ti_4_O_12_ ceramics. According to Fig. 5(a), the *τ*_1_ value of all of the samples remained almost constant. The *τ*_2_ and *I*_2_ values, as well as *τ*_ave_, continuously increased with the increase of Na^+^ doping content. This indicated that the size and number of cation vacancies in the doped samples increased. According to formula (2), Na^+^ did not increase the cation vacancy number. Thus, the increase in the amount of cation vacancies could be mainly associated with the volatilization of Na in the sintering process. Jumpatam *et al.* also reported the increase of vacancies caused by Na volatilization, which inhibited grain growth in Na_1/3_Ca_1/3_Y_1/3_Cu_3_Ti_4_O_12_ ceramics.^43^ With the increase of Na^+^ doping, the vacancy concentration continued to increase. This caused the cell shrinkage, which gradually counteracted the lattice expansion due to the presence of the Na^+^ dopant with the larger ion radius, eventually resulting in a smaller cell parameter for the sample with *x* = 0.08. This agreed with the XRD results depicted in Table 1. These cation vacancies inhibited the grain boundary migration and slowed down the grain growth.^6,41^ Thus, it can be concluded that the introduction of Na with the low melting point, along with the formation of the CuO phase and oxygen vacancies, was the main reason for the rapid grain growth at the Na^+^ doping amount less than 0.04. Meanwhile, the cation vacancies and the second phase containing Na inhibited grain growth at the doping amount above 0.04. Thus, the sample with *x* = 0.04 exhibited the largest grain size.

**Table 3:** Positron lifetime spectroscopy parameters for Cd_1−*x*_Na_*x*_Cu_3_Ti_4_O_12_ ceramics

Samples	*τ*_1_ (ps)	*τ*_2_ (ps)	*τ*_ave_ (ps)	*I*_1_ (%)	*I*_2_ (%)
*x* = 0.0	178.5	251.7	188.4	86.53	13.47
*x* = 0.02	177.3	254.0	189.4	84.21	15.79
*x* = 0.04	179.2	259.3	193.0	79.54	19.46
*x* = 0.06	178.5	266.4	200.4	75.02	24.98
*x* = 0.08	178.9	278.7	209.3	69.46	30.54

**Fig. 6:**
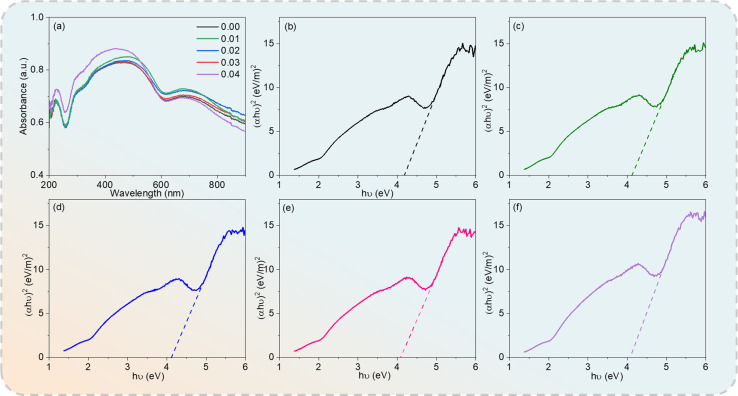
(a) UV-vis absorption spectra of Cd_1−*x*_Na_*x*_Cu_3_Ti_4_O_12_ ceramics and (*αhν*)^2^*versus h*ν plots at (b) *x* = 0.0; (c) *x* = 0.02; (d) *x* = 0.04; (e) *x* = 0.06; (f) *x* = 0.08.

UV-visible absorption spectroscopy was used to assess the optical properties and energy structure of the Cd_1−*x*_Na_*x*_Cu_3_Ti_4_O_12_ ceramics. Fig. 6(a) depicts the UV-vis absorption spectra of all of the specimens in the wavelength range of 200–900 nm. The optical energy band (*E*_g_) values were obtained using the Tauc method, as follows:^44^

3 (*αhν*)^2^ = *k*(*hν* − *E*_g_)

where *hν*, *α*, and *k* are the photon energy, the absorption coefficient, and a constant denoting the band edge parameter, respectively. The *E*_g_ values determined by the *X*-intercept of the tangent to the curve in Fig. 6(b–f) were 4.18, 4.13, 4.11, 4.08, and 4.06 eV for *x* = 0, 0.02, 0.05, 0.08, and 0.10, respectively, which were comparable with those of other ACTO-type ceramics.^8,45,46^ Na^+^ doping leads to an increase in the concentration of vacancies, thereby modulating the band structure and reducing the optical energy gap.^22,23^ Therefore, these wide band-gap materials have great application potential in high-performance optoelectronic and electronic devices.^47,48^

**Fig. 7:**
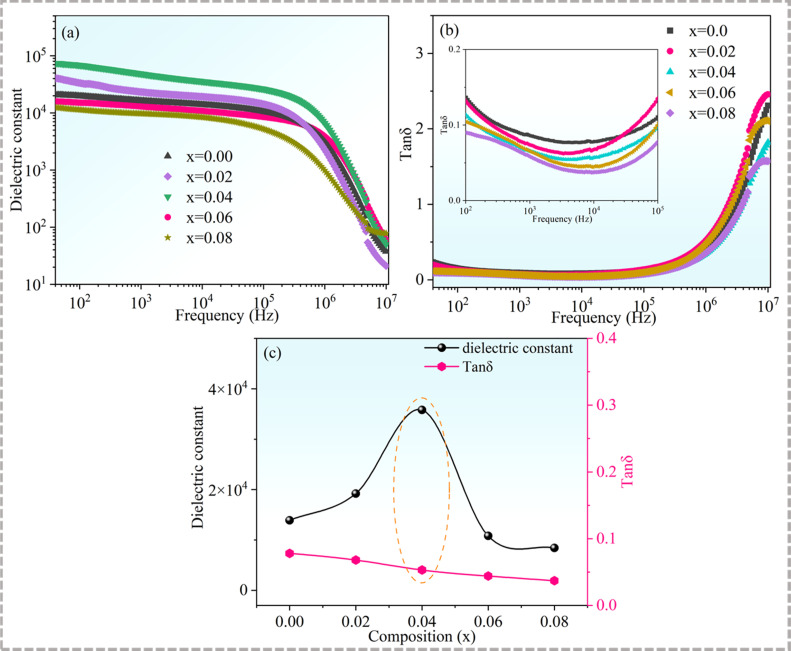
(a) Dielectric constant and (b) dielectric loss of Cd_1−*x*_Na_*x*_Cu_3_Ti_4_O_12_ ceramics as functions of frequency; the inset presents the magnified view in the frequency range from 100 to 10^5^ Hz; (c) dielectric constant and dielectric loss as a function of Na^+^ contents at 10 kHz.

Fig. 7 displays the dependences of the dielectric constants and dielectric losses of Cd_1−*x*_Na_*x*_Cu_3_Ti_4_O_12_ on the frequency. In general, the dielectric constants of all of the samples exhibited a plateau below 10^6^ Hz, indicating good frequency stability. It then decreased sharply above 10^6^ Hz, whereas a rapid increase of the dielectric loss occurred, which indicated a typical Maxwell–Wagner relaxation behavior.^49^ With the increase of Na^+^ doping content, the dielectric constant increased first and then decreased. According to the IBLC model, the dielectric constant is directly proportional to the grain size.^50^ The findings of the present study showed that the grain size and dielectric constant of the doped samples followed this relationship. The dielectric constants for Cd_1−*x*_Na_*x*_Cu_3_Ti_4_O_12_ ceramics with *x* = 0, 0.02, 0.04, 0.06, and 0.08 at 10 kHz were 13 900, 19 200, 35 800, 10 800, and 8400, respectively. Na^+^ doping also affected the dielectric loss of the samples. As seen from Fig. 7(b), the dielectric loss decreased with the increase of Na^+^ doping content. The corresponding values at 10 kHz for the Cd_1−*x*_Na_*x*_Cu_3_Ti_4_O_12_ ceramics with *x* = 0, 0.02, 0.04, 0.06, and 0.08 were 0.078, 0.068, 0.053, 0.044, and 0.037, respectively. The dielectric constant of the sample with *x* = 0.04 increased by two and a half times (from 13 900 to 35 800) relative to that of the pure CdCTO, while the dielectric loss decreased to a small extent (from 0.078 to 0.053). Fig. 7(c) depicts the variation of the dielectric constant and dielectric loss at 10 kHz with the Na^+^ content. It was found that the sample with *x* = 0.04 had an optimal dielectric property. That is, sodium doping can not only reduce the dielectric loss but also increase the dielectric constant, which is consistent with the results achieved in the Na^+^-doped CCTO ceramics.^39^

**Fig. 8:**
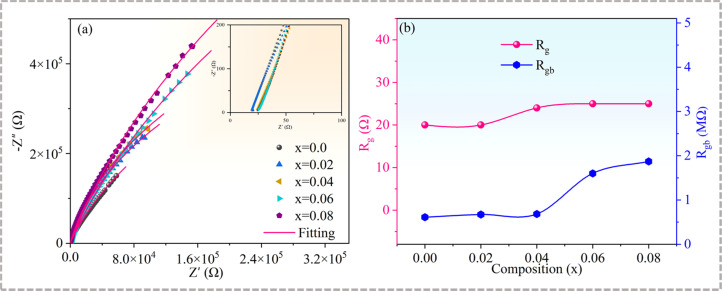
(a) Complex impedance plots of Cd_1−*x*_Na_*x*_Cu_3_Ti_4_O_12_ ceramics at room temperature; the inset shows an expanded view of the high-frequency data close to the origin; (b) *R*_g_ and *R*_gb_ parameters as functions of *x*.

The complex impedance characteristics at room temperature were determined to elucidate the reasons for the variation of the dielectric properties of the Cd_1−*x*_Na_*x*_Cu_3_Ti_4_O_12_ ceramics. Single semicircles with nonzero high-frequency intercepts were obtained (see Fig. 8 and the inset), where the low-frequency arc corresponded to the grain response and the high-frequency arc stood for the grain boundary response, respectively. An equivalent circuit model consisting of two parallel RC elements is usually used to simulate the impedance parameters. The grain resistance (*R*_g_) and grain boundary resistance (*R*_gb_) values of all samples, obtained using the ZsimpWin Version software, are presented in Fig. 8(b). The *R*_g_ value increased slightly in the range of 20 to 25 Ω, while the *R*_gb_ value dramatically increased with the increase of Na^+^ doping content. Semiconducting grains and insulating grain boundaries confirmed the electrical heterogeneity of all the samples. It is known that the lattice distortion resulting from the substitution ions with larger radii may promote the formation of a Cu-rich grain boundary layer, which can enhance the resistance of grain boundaries.^23,30^ Therefore, compared with the pure CdCTO, the specimens with *x* = 0.02 and 0.04 had higher *R*_gb_ values. The increase of *R*_gb_ caused by Na^+^ doping has also been reported in CCTO ceramics.^39^ In turn, the grain refinement increasing the number of grain boundaries has also caused the increase in *R*_gb_ at *x* = 0.06 and 0.08. Generally, the higher the grain boundary resistance is, the smaller the dielectric loss.^3,6–10^ The highest *R*_gb_ value obtained at *x* = 0.08 corresponded to the lowest tan *δ* (∼0.037), while the lowest *R*_gb_ value of the CdCTO sample yielded the highest tan *δ* value (∼0.078) at 10 kHz.

**Fig. 9:**
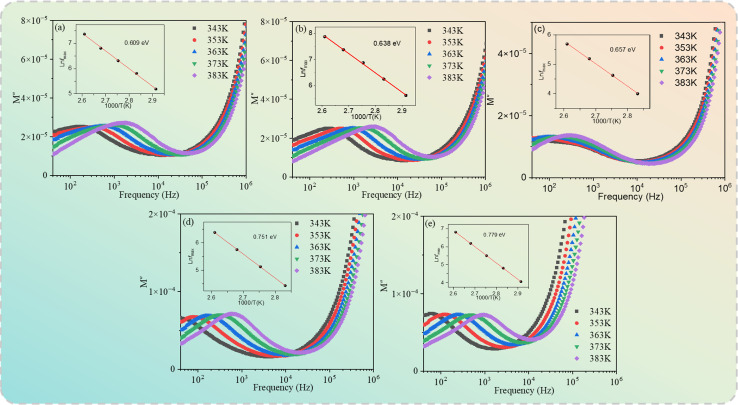
Electric modulus (*M*′′) as a function of the frequency of Cd_1−*x*_Na_*x*_Cu_3_Ti_4_O_12_ ceramics: (a) *x* = 0, (b) *x* = 0.02, (c) *x* = 0.04, (d) *x* = 0.06, and (e) *x* = 0.08.

The dielectric function of the electric modulus was used to establish the mechanism of the dielectric response of the samples. The complex modulus M* can be expressed as follows:^51^4
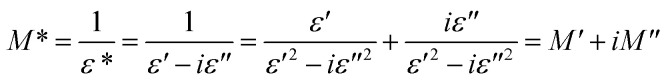
where ε* is a complex dielectric constant; and *M*′ and *M*′′ are the real and imaginary parts of the complex modulus, respectively. Fig. 9 depicts the frequency dependence of the electric modulus of the Cd_1−*x*_Na_*x*_Cu_3_Ti_4_O_12_ ceramics in the temperature range of 343−383 K. As seen from Fig. 9, only a set of modulus peaks emerged in the frequency range of 40–10^5^ Hz. For the samples with *x* = 0.04 and 0.06, the modulus peaks moved out of the measuring frequency window at 343 K. Moreover, the electric modulus peaks shifted to higher frequencies with the increase of temperature, indicating a thermally activated mechanism in all of the specimens. The frequencies and temperatures of the modulus peaks follow the Arrhenius law:^52^5
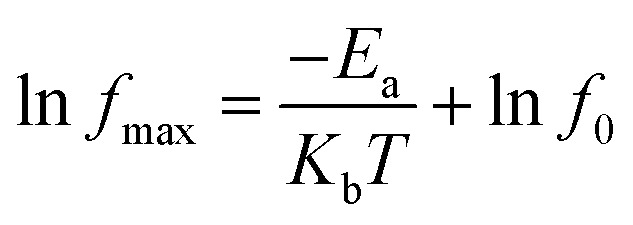
where *f*_max_, *f*_0_, *E*_a_, *k*_b_, and *T* are the peak frequency, the pre-exponential factor, the activation energy, the Boltzmann constant, and the temperature, respectively. The electric modulus peaks of all of the samples are clearly identified in Fig. 9. The inset of Fig. 9 shows the ln *f*_max_*versus* 1000/*T* and the plots obtained by fitting using eqn (5). The *E*_a_ values for the Cd_1−*x*_Na_*x*_Cu_3_Ti_4_O_12_ ceramics were 0.609, 0.638, 0.657, 0.751, and 0.779 eV, respectively. These values were consistent with the grain boundary conductivity activation energy of the CdCTO ceramics obtained in ref. 17–19, confirming that the dielectric relaxation originated from grain boundaries. Therefore, it was concluded that the dielectric constant of the Cd_1−*x*_Na_*x*_Cu_3_Ti_4_O_12_ ceramics at low frequency was due to the Maxwell–Wagner relaxation in relation to grain boundaries.

**Fig. 10:**
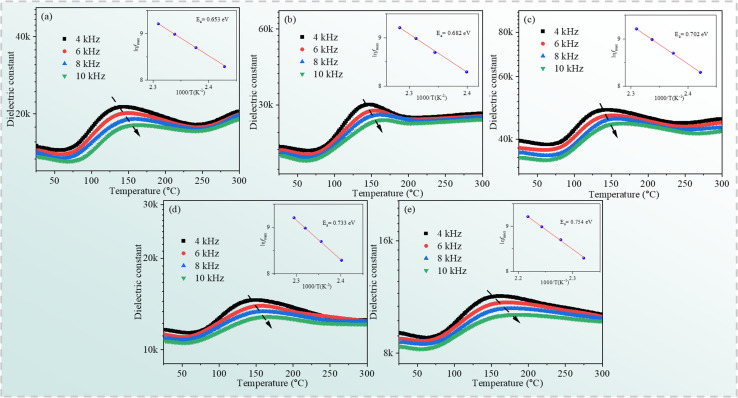
Temperature dependence of the dielectric constant for the Cd_1−*x*_Na_*x*_Cu_3_Ti_4_O_12_ ceramics (a) *x* = 0.0; (b) *x* = 0.02; (c) *x* = 0.04; (d) *x* = 0.06; (e) *x* = 0.08.

To further study the thermally activated mechanism in the specimens, the temperature dependence of the dielectric constant for the Cd_1−*x*_Na_*x*_Cu_3_Ti_4_O_12_ ceramics at 4, 6, 8, and 10 kHz is shown in Fig. 10. A set of the dielectric peaks emerged in the temperature range from 70 °C to 200 °C. The position of the peak shifted to higher temperature with the increase of frequency, while the peak intensity decreased in the specimens. The inset of Fig. 10 depicts the ln *f*_max_*versus* 1000/*T* plots fitted using eqn (5). The activation energy *E*_a_ values for the Cd_1−*x*_Na_*x*_Cu_3_Ti_4_O_12_ ceramics with *x* = 0, 0.02, 0.04, 0.06, and 0.08 were 0.653, 0.682, 0.702, 0.733, and 0.754 eV, respectively, which were related to the oxygen vacancies and similar to the values reported in ref. 17, 19, 23 and 24. In addition, the relaxation activation energy was depressed by Na doping.

**Fig. 11:**
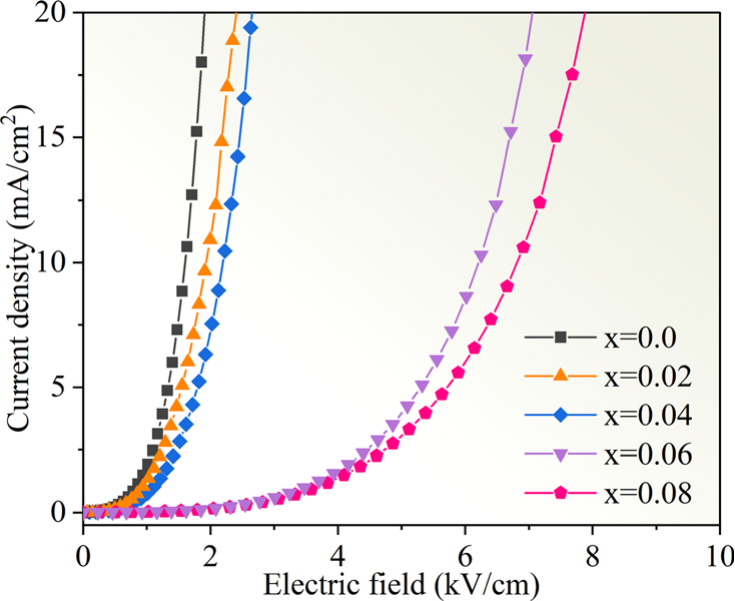
Nonlinear *J*–*E* plots of the Cd_1−*x*_Na_*x*_Cu_3_Ti_4_O_12_ ceramics.

**Fig. 12:**
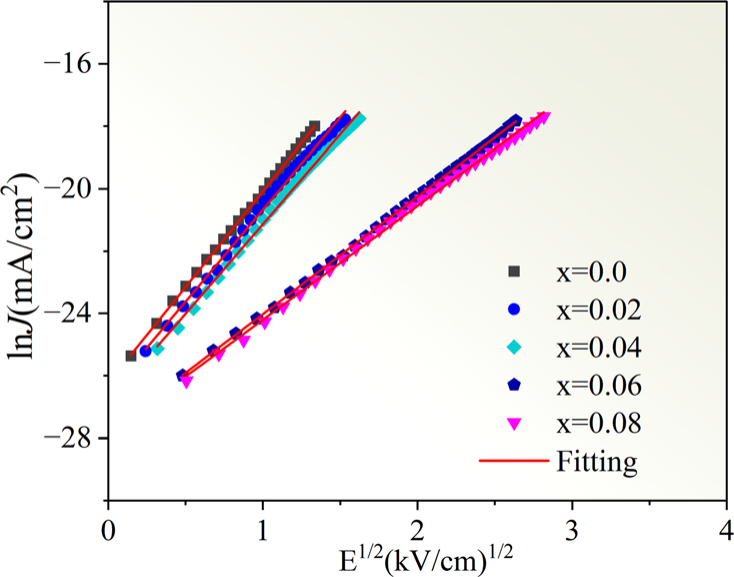
The ln *J versus E*^1/2^ plots for the Cd_1−*x*_Na_*x*_Cu_3_Ti_4_O_12_ ceramics.

Fig. 11 displays the nonlinear current density–electric field (*J*–*E*) plots of the Cd_1−*x*_Na_*x*_Cu_3_Ti_4_O_12_ ceramics. All samples exhibited good nonlinear characteristics. The corresponding *α* and *E*_b_ values were found to be 2.53, 2.99, 2.85, 3.04, 3.66, and 0.82, 0.93, 1.1, 3.47, and 3.82 kV cm^−1^ at *x* = 0, 0.02, 0.04, 0.06, and 0.08, respectively, surpassing those reported in ref. 20 and 21. For electrically heterogeneous ACTO materials, the nonlinear electrical properties usually originate from the Schottky barriers between the semiconducting grains and insulating grain boundaries.^7,9,12,13^ According to the Schottky model, the grain boundary barrier (*Φ*_b_) can be obtained from the relationship between *J* and *E* as follows:^53^7
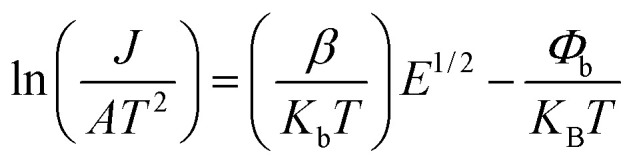
where *A*, *β*, *k*_b_, and *T* are the Richardson's constant, the constant related to the potential barrier width, the Boltzmann constant, and the temperature, respectively. Fig. 12 depicts the ln *J versus E*^1/2^ plots for the Cd_1−*x*_Na_*x*_Cu_3_Ti_4_O_12_ ceramics, which exhibited a good linear relationship and confirmed the existence of the Schottky barriers in all of the samples. According to the fitting results in Fig. 12, the barrier heights (*Φ*_b_) were 0.679, 0.688, 0.708, 0.719, and 0.722 eV, respectively, at *x* = 0, 0.02, 0.04, 0.06, and 0.08. These results were close to the *E*_gb_ values of the CCTO ceramics.^6,41^ The variation of the barrier height was consistent with the grain boundary resistance and the activation energy *E*_a_. It is worth noting that the sample with *x* = 0.08 possessed the optimal nonlinear properties due to its highest grain boundary resistance and barrier height. In addition, these *Φ*_b_ values were comparable with the above activation energies *E*_a_, which confirmed that the dielectric relaxation in the low-frequency range originated from grain boundaries. To analyze the Na^+^ doping effect on the dielectric properties and nonlinear response of the CdCTO specimens, a comparison of the *ε*′, tan *δ*, *α*, and *E*_b_ values of the Cd_1−*x*_Na_*x*_Cu_3_Ti_4_O_12_ ceramics with previously reported values in the literature is summarized in Table 4. It is obvious that our Cd_1−*x*_Na_*x*_Cu_3_Ti_4_O_12_ ceramics exhibited greater dielectric and nonlinear properties than the ACTO-based ceramics.

**Table 4:** Comparison of dielectric constant (*ε*′) and dielectric loss (tan *δ*) at 1 or 10 kHz, the nonlinear coefficient (*α*) and breakdown field strength (*E*_b_) of the Cd_1−*x*_Na_*x*_Cu_3_Ti_4_O_12_ ceramics with previously reported values in the literature

Samples	*ε*′	tan *δ*	*α*	*E*_b_	Ref.
CdCTO	2.4 × 10^4^	0.072	—	—	54
Ca_0.98_Na_0.02_Cu_3_Ti_4_O_12_	9.8 × 10^4^	0.073	—	—	22
CdMg_0.1_Cu_2.9_Ti_4_O_12_	5.0 × 10^4^	0.1	—	—	17
Na_0.35_Bi_0.55_Cu_3_Ti_4_O_12_	0.76 × 10^4^	0.015	—	—	24
CdCu_2.9_Zn_0.1_Ti_4_O_12_	4.0 × 10^4^	0.058	—	—	18
Na_0.5_La_0.5_Cu_3_Ti_4_O_12_	1.5 × 10^4^	0.047	—	—	25
Na_0.05_Y_0.65_Cu_3_Ti_4_O_12_	0.75 × 10^4^	0.022	—	—	23
CdCTO-2.0wt%SiO_2_	0.52 × 10^4^	0.06	—	1.90	21
CdCTO-3wt%Al_2_O_3_	1.0 × 10^4^	0.1	4.89	1.78	20
Cd_0.96_Na_0.04_Cu_3_Ti_4_O_12_	3.4 × 10^4^	0.053	2.85	1.10	This work
Cd_0.94_Na_0.06_Cu_3_Ti_4_O_12_	1.1 × 10^4^	0.044	3.04	3.47	This work
Cd_0.92_Na_0.08_Cu_3_Ti_4_O_12_	0.84 × 10^4^	0.037	3.66	3.82	This work

## 4. Conclusions

In this study, Cd_1−*x*_Na_*x*_Cu_3_Ti_4_O_12_ ceramics were prepared *via* the solid-state reaction method. Their phase composition, microstructure, and defect characteristics, as well as the optical, dielectric, and nonlinear properties were systematically investigated. In addition to the main Cd_1−*x*_Na_*x*_Cu_3_Ti_4_O_12_ phase, the CuO second phase was found in the doped samples. The number of cation vacancies increased with the increase in Na^+^ doping content. Na with the low melting point, along with the formation of CuO phase and oxygen vacancies, was the main reason for the rapid grain growth at the Na^+^ doping amount of less than 0.04. Meanwhile, the cation vacancies and the second phase containing Na inhibited grain growth at the doping amount above 0.04. The optical energy band decreased with the multiplication of cation vacancies. The dielectric constant increased with the increase of grain size and followed the IBLC effect. The sample with *x* = 0.04 exhibited the highest *ε*′ value of ∼35 800 and a lower tan *δ* of ∼0.053 at 10 kHz. The lowest tan *δ* of ∼0.037, along with the optimal nonlinear *α* and *E*_b_ values of ∼3.21 and ∼2.47 kV cm^−1^, respectively, was achieved in the sample with *x* = 0.08, which was associated with its highest grain boundary resistance and barrier height. Therefore, the electric modulus data proved that the large dielectric constant of the Cd_1−*x*_Na_*x*_Cu_3_Ti_4_O_1_ ceramics was ascribed to the Maxwell–Wagner effect at the grain boundaries.

## Data availability

The data that support the findings of this study are available from the corresponding author upon reasonable request.

## Conflicts of interest

No conflict of interest exists.
